# RETRACTION: Comparative efficacy of semaglutide versus liraglutide on weight loss and glycaemic control

**DOI:** 10.1530/EC-25-0723r

**Published:** 2026-03-06

**Authors:** Hashmat Ullah, Zahid Ullah Khan, Asif Wazir, Usman Khan, Anees Ur Rehman, Peer Shoaib, Muhammad Arsalan Sharif

**Affiliations:** ^1^ Department of Endocrinology, Lady Reading Hospital, Peshawar, Pakistan; ^2^ Department of Medicine, Lady Reading Hospital, Peshawar, Pakistan; ^3^ Emergency Medicine Department, Lady Reading Hospital, Peshawar, Pakistan; ^4^ Mayo Hospital, Urology, Lahore, Pakistan

This article has been retracted by the journal. The retraction was made before the article reached its final form in the publication process. However, the authors’ accepted manuscript, prior to copy editing, page layout and proofing, was made available online as an *Endocrine Connections* Accepted Manuscript on 14 January 2026.

The article has been retracted because *Endocrine Connections* developed serious concerns about the role of generative AI in preparing the manuscript. The authors made no declaration of the use of generative AI on submission of the manuscript, or during review, in contravention of the journal’s AI policy. In addition, the journal developed serious concerns related to the data presented in Figs 1 and 2. The authors supplied additional data in response to the concerns raised by the journal, in which further inconsistencies were evident.

As such, the article has now been retracted. No further action will be taken against the authors of the article.

This retraction was published on 6 March 2026.

